# Knowledge of breast cancer, risk factors and symptoms among males and females in Botswana

**DOI:** 10.4102/curationis.v49i1.2811

**Published:** 2026-10-07

**Authors:** Punishment P. Chibatamoto, Mpho Keetile, Moses J. Chimbari

**Affiliations:** 1 Discipline of Public Health, College of Health Sciences, University of KwaZulu-Natal, Durban, South Africa; 2 Department of Population Studies, Faculty of Social Science, University of Botswana, Gaborone, Botswana

**Keywords:** Botswana, breast cancer, knowledge, risk factors, symptom, male, female, public health education

## Abstract

**Background:**

Breast cancer is a major cause of death among African females, often diagnosed late due to limited knowledge about the disease. In Botswana, where late-stage presentation is common, there is limited information on the knowledge levels of breast cancer, its risk factors and symptoms among both male and female.

**Objectives:**

This study aimed to assess knowledge levels of breast cancer, its risk factors and symptoms among both male and female in Botswana.

**Method:**

A cross-sectional survey collected demographic and breast cancer knowledge data from adult male and female patients in public hospital outpatient departments of Botswana using a structured questionnaire.

**Results:**

Most (69.3%) of the 228 participants were female. Most (90.5% female, 82.9% male) viewed breast cancer as a serious illness, 95.6% hereditary (96.2% female, 94.3% male) and 88.6% (90% male, 87.9% female) recognised the importance of early detection. Less than 15% of all respondents correctly identified breast cancer risk factors. Odds of knowledge about breast cancer (Adjusted Odds Ratio [AOR] = 1.31, Confidence Interval [CI] = 1.01–2.78), symptoms (AOR = 4.29, CI = 1.28–6.72) and risk factors (AOR = 1.17, CI = 1.02–2.34) were significantly higher among female participants than male participants.

**Conclusion:**

There is a need for health educational programmes tailored to specific demographics, including both male and female, that address key risk factors and symptoms for enhanced breast cancer awareness and early detection.

**Contribution:**

Our study adds to the literature on the importance of shifting from general awareness campaigns to targeted educational initiatives that equip individuals with the knowledge and tools to make informed decisions about their health and seek timely medical care when needed.

## Introduction

Breast cancer represents a significant global health issue, ranking as the second most common cancer worldwide with 2.3 million new cases reported, following lung cancer (World Health Organization [WHO] 2024). It is also the fourth leading cause of cancer-related mortality, with 685 000 deaths among females documented in 2022 (WHO 2024). The WHO indicates that although breast cancer affects both genders, it disproportionately impacts females, who account for about 99% of cases globally, making being female the most significant risk factor. Other risk factors include genetic predispositions, age, family history, lifestyle, hormonal influences, reproductive history, and environmental factors (Obeagu & Obeagu 2024; Sun et al. 2017).

Since 2000, developed countries have seen a decline in breast cancer incidence and mortality rates due to increased awareness, widespread screening, early detection, and comprehensive treatment programmes (DeSantis et al. 2016). Conversely, developing nations are experiencing a rise in breast cancer cases, with it being the second leading cause of cancer-related deaths among females in Africa (Bray et al. 2024; Francies et al. 2020). Africa’s challenges in breast cancer management include limited healthcare access, inadequate diagnostic facilities, and insufficient screening programmes, resulting in higher mortality rates (Anyigba, Awandare & Paemka 2021). These issues are exacerbated by high treatment costs, unequal healthcare access, and a lack of awareness campaigns (Anyigba et al. 2021; WHO 2023).

In sub-Saharan Africa, breast cancer is often diagnosed at advanced stages, notably in Zimbabwe, at >80% in 2023 (Magara et al. 2023), Tanzania, at 80% in 2022 (Sakafu et al. 2022), Kenya, at 80% in 2022 (Kailemia 2022), Zambia, at 66% in 2020 (Songiso et al. 2020) and South Africa, at 63.3% in 2022 (Anyigba et al. 2021; WHO 2024). Botswana, similarly affected, reported 257 new breast cancer cases (11.1% of all new cases) and 91 deaths (7.1% of all cancer-related deaths among female) (Ferlay 2024). Breast cancer is the second most common cancer among females in Botswana, following cervical cancer, with about 65% of cases diagnosed at advanced stages in 2022, compared to only 13% in the United States (Sinha et al. 2022).

Studies reveal that a lack of knowledge regarding breast cancer, its risk factors, and symptoms significantly contributes to late-stage diagnoses (Tesfaw, Alebachew & Tiruneh 2020). In developing regions, many females delay seeking medical care due to a lack of awareness about early symptoms, often mistaking them for less serious conditions. This delay can lead to advanced disease stages, limiting treatment options and adversely affecting outcomes. Therefore, enhancing knowledge about breast cancer is critical for early detection and treatment, empowering individuals to recognise potential issues, seek timely medical care, and reduce disease-associated stigma and fear.

Understanding knowledge levels is important for developing targeted interventions, such as education and screening programmes, to improve early detection and reduce mortality rates. Educational initiatives have been shown to significantly increase awareness of breast cancer and the importance of early detection (Mehiret, Molla & Tesfaw 2022; Nisha & Murali 2020). Knowledge of risk factors, such as family and personal history, is also crucial. Research indicates that female with a family history of breast cancer are more informed about various risk factors (Endrikat et al. 2024). Tailoring interventions to specific populations and addressing barriers to knowledge and behaviour change are essential, as studies have shown that educational programmes significantly enhance understanding of breast cancer symptoms, risk factors, treatments, and prevention measures among female students (Sarker et al. 2022).

Assessing breast cancer knowledge in Botswana provides valuable insights into the specific needs and challenges of the country’s citizenry. This facilitates the development of targeted culturally appropriate interventions that enhance early screening rates, reduce stigma and empower individuals to make informed breast health decisions. However, there is limited information on breast cancer awareness and knowledge levels in Botswana, as existing research primarily focuses on females and is concentrated in the capital city (Mbuka-Ongona & Tumbo 2013; Tieng’o et al. 2011).

Although breast cancer is less common in males, educating them about the risk is important, as well as encouraging them to support female relatives and partners in seeking timely healthcare. This study addresses a significant gap in knowledge about breast cancer, its risk factors, and symptoms among both males and females in Botswana, where information is currently scarce. Our aim was to assess the general knowledge levels of breast cancer, risk factors and symptoms among both genders in Botswana.

## Research methods and design

### Study design

We conducted a cross-sectional survey among adults (both male and female aged 18 years and older) attending outpatient departments in four public district hospitals. These hospitals were strategically selected to represent diverse regions of the country. The cross-sectional design allowed us to gather data from eligible participants at a single point in time, evaluating their knowledge of breast cancer, its risk factors, and symptoms (Greenwood & Levin 2006).

### Study setting

The selected public district hospitals offer a wide range of health services, including free oncology-related services. A typical public district hospital comprises outpatient and specialised departments. Outpatient departments handle general consultations, preventive services, health promotion, rehabilitative services, and treatment for common illnesses and injuries. Specialised departments include maternity wards, surgical units, infectious disease control centres, eye and dental clinics, and clinical support services. The hospitals chosen are in Francistown (Northern Region) with a population of 103 417, Maun (Northwestern Region) serving 85 350 people, Serowe (Central Region) serving 55 676 people, and Goodhope (Southern Region) serving 5672 people for a country with a population of 2 359 609 (Statistics Botswana 2022). At the time of the study, Botswana had an extensive healthcare network comprising three national referral hospitals, 15 district hospitals, 17 primary hospitals, 105 clinics with beds, 206 clinics without beds, 351 health posts, and 931 mobile stops across 27 health districts.

### Study population and sampling strategy

The study targeted adult male and female aged 18 years and older, who were citizens of Botswana and attended outpatient departments at the four participating hospitals. Exclusions were made for critically ill patients, those with learning difficulties, communication barriers, and those accompanying patients. Historical outpatient attendance data from the participating facilities indicated an estimated monthly average of 200 adult patients (70% female, 30% male) meeting the eligibility criteria. Assuming a sufficiently large number of eligible patients, we calculated sample sizes of 140 females (200*0.70) and 60 males (200*0.30) as shown below.

A stratified sampling design with proportional allocation was applied to determine the sub-sample sizes for each gender (male and female). After establishing these sizes, systematic sampling to select individuals from each group was applied. The sample size was determined through a four-step process. An initial calculation used Cochran’s formula with a 95% confidence level, a 5% margin of error, and a 50% knowledge estimate for a country with a population of 2 359 609 using the following formula in Equation 1:


$$n_{0}=\frac{Z^{2}\cdot p(1-p)}{d^{2}}=\frac{1.96^{2}\cdot 0.5(1-0.5)}{0.05^{2}}=384.16$$ [Eqn 1]

Where:

- *Z* = The standard normal deviate for the desired confidence level (typically 1.96 for a 95% confidence level).
- *p* = The estimated proportion of knowledge in the population. If unknown, a value of 0.5 (50%) is used to maximise the sample size for a conservative estimate.
- *d* = The desired margin of error or precision level (e.g. 0.05 for ± 5%).

This was then adjusted using a finite population correction (FPC) due to a limited population of 200 eligible patients per month determined from the historical outpatient attendance data. The FPC formula is shown in Equation 2:


$$n_{adj}=\frac{n_{0}}{1+\frac{n_{0}-1}{N}}=\frac{384.16}{1+\frac{384.16-1}{200}}=131.75$$ [Eqn 2]

Where:

- *N* = Total population size of 200 adult patients (70% female, 30% male) in a month based on historical data.
- *n*_*adj*_ = The final adjusted sample size.

As the adjusted FPC sample size (132) is smaller than the monthly historical outpatient departments sample, 200 was stratified by gender (70% female, 30% male) for proportional allocation for each gender. The formula for each stratum (*n*_*h*_) applied can be seen in Equation 3:


$$n_{h}=N\times P_{h}$$ [Eqn 3]

Where:

- *n*_*ℎ*_ = Sample size for the specific stratum (70% female and 30% male)
- *N* = Total estimated population (average of 200 in this study).
- *P_ℎ_* = Proportion of the population belonging to that stratum (70% for females; 30% for males).
- *n_female_* = 0.70 × 200 = 140
- *n_male_* = 0.30 × 200 = 60

The adjusted sample was stratified by gender (70% female, 30% male), resulting in targets of 140 females and 60 males, who were then selected using a systematic sampling interval computed using the formula in Equation 4:


$$k=\frac{N}{n}$$ [Eqn 4]

Where:

- *k* = Sampling interval (the ‘skip’ or every *K*^th^ person).
- *N* = Total population available for that stratum.
- *n* = Desired sample size for that stratum.

Therefore, for female, *k* = 200/140≈1.43 and for male, *k* = 200/60≈3.33. Based on these intervals, every second female and every fourth male were selected to create a representative sample, which was deemed feasible during pre-testing, which was conducted in a non-participating health facility.

### Data collection process

Structured questionnaires, adapted from the Breast Cancer Initiative (Dvaladze et al. 2016), were developed separately for males and females, covering sociodemographic characteristics, breast cancer knowledge, and awareness of risk factors and symptoms. The tool consisted of multiple-choice questions and true or false or don’t know options. The questionnaire underwent face and content validation by local experts and was translated into Setswana with back-translation to ensure conceptual equivalence. The tool was pre-tested on 20 participants (10 female and 10 male) at a non-participating hospital and confirmed validity, reliability, and internal consistency. The pre-test confirmed that the questionnaire effectively captured the intended responses, with an average interview time of 45 min per person. The pre-test data from these 20 individuals were not included in the main study analysis. The finalised questionnaire was administered to eligible participants as they exited outpatient consultation rooms from 01 October to 31 October 2023 (Monday–Friday) between 08:00 and 13:00 at each participating facility.

### Data analysis

Data were entered into Microsoft Excel and manually verified against raw data from the questionnaires. The final dataset was exported to Stata 18 for analysis. Sociodemographic characteristics of participants were summarised using descriptive statistics, stratified by gender, and presented as frequency distributions. Chi-squared tests and Fisher’s exact tests (where applicable) compared responses between male and female, with a *p*-value of < 0.05 considered statistically significant. Multivariable logistic analysis was conducted to explore associations, with variables showing a *p*-value < 0.2 in univariate analysis included in the multivariate model (Yano et al. 1997). Model goodness of fit was tested using the Hosmer–Lemeshow test and link test (Hosmer et al. 1997).

### Ethical considerations

Ethical approval was obtained from the Health Research Development Committee (HRDC) of the Ministry of Health, Botswana (reference number HPDME 13/18/1), and the Biomedical Research Ethics Committee at the University of KwaZulu-Natal, South Africa (reference number BREC/00001596/2020). Participants were informed that the study posed no physical, psychological, social, or economic risks and that their participation would contribute to improved breast cancer management knowledge and innovations. Informed consent was obtained, and participants were assured of their autonomy to withdraw from the study if they chose not to participate. Written consent was secured before enrolling participants in the study.

## Results

### Sociodemographic characteristics

The study sample comprised 228 adults, with a majority being female, 69.3% (*n* = 158), and 30.7% (*n* = 70) male (Table 1). The mean age of male participants was slightly higher (41.07 ± 13.42 years, ranging from 19 years to 74 years) than that of female participants (38.71 ± 13.20 years, ranging from 18 years to 82 years). The highest proportion of participants was aged 25 years – 34 years (30.7% male and 28.9% female). A high percentage of male (62.3%) and female (71.5%) were single.

**TABLE 1:** Socio-demographic characteristics of study participants (*N* = 228).

Socio-demographic variables	Male		Female		Total (*N*)
	*n*	%	*n*	%	
**Age (years)**					
18–24	4	05.7	23	14.6	27
25–34	22	30.7	45	28.9	67
35–44	17	24.3	43	27.4	60
44–54	12	17.1	30	19.1	42
55–64	11	15.1	7	04.5	18
≥ 65	3	04.3	9	05.7	12
**Marital status**					
Cohabiting	1	01.4	6	03.8	7
Divorced	1	01.4	5	03.2	6
Married	21	30.0	28	17.7	49
Other	0	00.0	2	01.3	2
Single	45	62.3	113	71.5	158
Widowed	2	02.9	4	02.5	6
**District**					
Francistown	15	21.4	36	22.8	51
Goodhope	20	28.6	45	28.5	65
Maun	17	24.3	52	32.9	69
Serowe	18	25.7	25	15.8	43
**Religion**					
None	22	31.4	26	16.5	48
Other	31	44.3	74	46.8	105
Pentecostal	7	10.0	31	19.6	38
Protestant	9	12.9	27	17.1	36
Traditional	1	01.3	0	00.0	1
**Education level**					
Completed primary	15	21.4	32	20.3	47
Completed secondary	13	18.6	37	23.4	50
Completed tertiary	13	18.6	29	18.4	42
None	7	10.0	4	02.5	11
Not completed primary	5	07.1	17	10.8	22
Not completed secondary	15	28.8	37	23.4	52
Not completed tertiary	1	33.3	2	01.3	3
Other	1	100	0	00.0	1
**Occupation**					
Clerical and/or management and/or administrative	14	20.0	23	14.6	37
Crafts	15	21.4	13	08.2	28
Farmer	6	08.6	3	01.9	9
Housewife	0	00.0	7	04.4	7
None	2	02.9	87	55.1	89
Other	29	41.4	15	9.51	44
Professional technical	4	05.7	2	01.3	6
Trader	0	00.0	8	05.1	8

**Total**	**70**	**-**	**158**	**-**	**228**

A high proportion of male participants were from Goodhope district, while females were mainly from Maun (32.9%). An almost equal number of males (44.3%) and female (46.8%) indicated that they belong to other religions. More males (*n* = 15) indicated that they completed primary education, while most females had completed secondary education (*n* = 37). Most males indicated that they were employed (41.4%), while most women reported that they were unemployed (55.4%).

### General knowledge about breast cancer

Our study revealed a general level of knowledge about breast cancer among participants, with statistically significant differences observed between genders. Almost all participants, excluding one man, reported having heard about breast cancer. A significant minority (30.7%) of respondents, with females being slightly more likely to report this at 32.9% and male at 25.1%, reported knowing a person with breast cancer (Table 2).

**TABLE 2:** Participants’ responses to general knowledge questions about breast cancer (*N* = 228).

General knowledge questions	Total				Male (*N* = 70)				Female (*N* = 158)				*p*-value*
	Yes		No or do not know		Yes		No or do not know		Yes		No or do not know		
	*n*	%	*n*	%	*n*	%	*n*	%	*n*	%	*n*	%	
Ever heard of BC?	227	99.6	1	00.4	69	98.6	1	01.4	158	100	0	00.0	< 0.001
Know a person with BC?	70	30.7	157	69.3	18	25.1	51	72.9	52	32.9	106	67.1	< 0.001
Is BC a serious illness?	201	88.2	27	11.8	58	82.9	12	17.1	143	90.5	15	09.5	< 0.001
BC is due to heredity?	218	95.6	10	04.4	66	94.3	4	05.7	152	96.2	8	03.8	< 0.001
BC is due to lifestyle?	12	05.3	216	94.7	6	08.5	64	91.5	6	03.8	152	96.2	< 0.001
Know the cause of BC?	18	07.9	210	92.1	1	01.4	69	98.6	21	13.3	137	86.7	< 0.001
Do males get BC?	110	48.2	118	51.8	32	45.7	38	54.3	78	49.4	80	50.6	< 0.001
Is BC curable?	136	59.6	92	40.4	42	60.0	28	40.0	94	59.5	64	40.5	< 0.001
Can one survive if BC is detected early?	202	88.6	56	24.6	63	90.0	7	10.0	139	87.9	19	12.1	< 0.001
Traditional healers can heal BC?	186	81.6	42	18.4	55	78.6	15	21.4	131	82.9	27	17.1	< 0.001

BC, breast cancer.

* , *p*-value of differences between male and female.

About half (48.2%) of respondents recognised that males can also get cancer. While 49.4% of females acknowledged this, 45.7% of males did. Most (88.2%) respondents (90.5% female and 82.9% male) considered breast cancer a serious illness in Botswana, and over half (59.6%) believed breast cancer could be cured, with males (60.0%) showing a slightly higher belief than females (59.5%). Furthermore, most (95.6%) of those surveyed believed breast cancer was hereditary (96.2% of female, 94.3% of male), and 88.6% knew that early detection could improve survival (90% of male, 87.9% of female).

While a minority of respondents (7.9%) indicated familiarity with breast cancer causes, female (13.3%) exhibited a higher rate of knowledge compared to male (1.4%). While 5.3% of all respondents attributed breast cancer to lifestyle factors, this belief was more prevalent among male (8.5%) than female (3.8%). Additionally, a significant proportion of both groups believed that traditional healers could heal breast cancer (78.6% male and 82.9% female). All these comparisons were statistically significant (*p* < 0.001).

### Knowledge about breast cancer risk factors

Participants were asked questions about breast cancer risk factors. A significantly high proportion of participants (96.1%) incorrectly indicated that advanced age is not a risk factor for breast cancer (Table 3). When this was disaggregated by gender, a higher proportion of females (97.4%) than males (92.9%) incorrectly responded to the question. Most study participants (96.1%) incorrectly believed poverty is not a breast cancer risk factor. This misconception was especially prevalent among females (97.4%) compared to males (92.9%), indicating that a large majority of people, particularly females, did not understand that poverty can be a risk factor for breast cancer.

**TABLE 3:** Participants’ responses to questions about breast cancer risk factors (*N* = 228).

Questions on risk factors for breast cancer	Total				Male (*N* = 70)				Female (*N* = 158)				*p*-value*
	Correct		Incorrect or do not know		Correct		Incorrect or do not know		Yes		Incorrect or do not know		
	*n*	%	*n*	%	*n*	%	*n*	%	*n*	%	*n*	%	
Is advanced age a risk factor?	9	03.9	219	96.1	5	07.1	65	92.9	4	02.5	154	97.4	< 0.001
Is poverty a risk factor?	9	03.9	219	96.1	5	07.1	65	92.9	4	02.5	154	97.4	< 0.001
Is wearing brassieres risk factor?	19	08.3	209	91.6	6	08.6	64	91.4	13	08.3	145	91.7	< 0.001
Is the first childbearing after 30 years risk factor?	5	02.2	223	97.8	3	04.3	67	95.7	2	01.3	156	98.7	< 0.001
Is obesity a risk factor?	19	08.3	209	91.7	11	15.7	59	84.3	8	05.1	150	94.9	< 0.001
Is contact with BC person a risk factor?	3	01.3	225	98.7	0	00.0	70	100	3	01.9	155	98.1	< 0.001
Is the first relative family history a risk factor?	25	10.9	203	89.1	9	12.9	61	87.1	16	10.1	142	89.9	< 0.001
Is a long lactating period a risk factor?	7	03.1	221	96.9	4	05.7	66	94.3	3	01.9	155	98.1	< 0.001
Is early menarche a risk factor?	5	02.3	225	98.7	2	02.9	68	97.1	3	01.9	155	98.1	< 0.001
Is late menopause a risk factor?	9	03.9	219	96.1	3	04.3	67	95.7	6	03.8	152	96.2	< 0.001
Is an oral contraceptive a risk factor?	4	01.8	224	98.2	2	02.9	68	97.1	2	01.0	226	99.0	< 0.001
Is tobacco a risk factor?	31	13.6	197	86.4	13	18.6	57	81.4	18	11.6	140	88.6	< 0.001
Is alcohol a risk factor?	28	12.3	200	87.7	11	15.7	59	84.3	17	10.8	111	70.3	< 0.001
Is a high-fat diet a risk factor?	20	08.8	208	91.2	10	14.3	60	85.7	10	06.3	148	93.4	< 0.001
Are big breasts a risk factor?	11	04.8	217	95.2	3	04.3	67	95.7	8	05.1	150	94.9	< 0.001

BC, breast cancer.

* , *p*-value of differences between male and female.

Most participants (97.8%) mentioned primiparity beyond age 30 as a breast cancer risk factor, a misconception that was marginally more prevalent among female respondents (98.7%). When asked if obesity is a risk factor for breast cancer, most participants either gave a wrong answer or said they did not know (91.7%), and this was highest among females (97.4%). Our study revealed that most participants (98.7%) demonstrated a lack of knowledge regarding the contagious nature of cancer, with this misconception being particularly pronounced among all male participants. Most (89.1%) participants were unaware that having a family history with first-degree relatives is a risk factor for breast cancer, with females (89.9%) being slightly more unaware.

Participants were also asked whether a long lactating period is a risk factor and whether early menarche is a risk factor for breast cancer. Most of them gave wrong answers or said they did not know (96.9% and 98.7% respectively), and the majority were among females (98.1%). On whether late menopause is a risk factor, most participants (96.1%) provided a wrong answer or said they did not know, with most of them being females (96.1%). A high proportion of participants responded incorrectly (or did not know) for all other questions (is an oral contraceptive a risk factor? [98.2%]; is tobacco a risk factor? [86.4%]; is alcohol a risk factor? [87.7%]; is a high-fat diet a risk factor? [91.2%] and are big breasts a risk factor? [95.2%]). The highest proportion was among females for all these questions except for the question ‘Is alcohol a risk factor?’ where most males (84.3%) than females (70.3%) responded incorrectly.

### Predictors of knowledge about breast cancer

The odds ratios for the likelihood of general knowledge about breast cancer, knowledge about breast cancer symptoms and knowledge about risk factors of breast cancer among participants were stratified by socio-demographic factors. The odds of general knowledge about breast cancer (Adjusted Odds Ratio [AOR] = 1.31, Confidence Interval [CI] = 1.01–2.78), knowledge about breast cancer symptoms (AOR = 4.29, CI = 1.28–6.72) and knowledge about risk factors for breast cancer (AOR = 1.17, CI = 1.02–2.34) were significantly higher among females than males (Table 4). For age, the odds of having correct knowledge about breast cancer were significantly higher among individuals aged 18 years – 24 years (AOR = 1.30, CI = 1.00–2.81) compared to those aged 65 years and above. The respondents who reported that they were in a cohabiting relationship (AOR = 1.95, CI = 1.15–3.33) were more likely to have knowledge about breast cancer symptoms than those who were widowed.

**TABLE 4:** Predictors of breast cancer knowledge among respondents.

Variable	Knowledge about BC		Knowledge about BC symptoms		Knowledge about risk factors of BC	
	AOR	95% CI	AOR	95% CI	AOR	95% CI
**Sex**						
Male	1.00	-	1.00	-	1.00	-
Female	1.31**	1.01–2.78	4.29**	1.28–6.72	1.17**	1.02–2.34
**Age (years)**						
18–24	1.30**	1.00–2.81	0.71	0.31–1.58	2.20	0.02–3.04
25–34	2.62	0.78–3.76	0.68	0.30–1.58	0.79	0.03–1.71
35–44	1.15	0.52–2.98	0.54	0.32–1.22	0.90	0.05–1.83
44–54	1.63	0.61–3.03	0.51	0.29–1.19	0.76	0.02–1.80
55–64	6.76	0.98–12.3	0.66	0.28–1.28	1.41	0.23–5.57
≥ 65	1.00	-	1.00	-	1.00	-
**Marital status**						
Cohabiting	3.07	0.81–6.14	1.95**	1.15–3.33	14.1	0.93–28.2
Divorced	2.04	0.58–4.08	14.4	0.06–33.3	0.01	0.00–1.11
Married	3.05	0.79–6.11	2.15	0.01–42.2	0.02	0.00–1.20
Other	1.02	0.32–2.04	1.58	0.58–3.16	0.30	0.00–0.60
Single	1.12	0.48–2.24	2.86	0.72–3.92	0.68	0.30–1.58
Widowed	1.00	-	1.00	-	1.00	1.00
**District**						
Francistown	2.73**	1.12–3.78	3.08**	1.52–7.16	2.40**	1.02–4.80
Goodhope	1.01	0.31–2.03	1.15	0.53–2.30	2.37**	1.01–4.74
Maun	1.18	0.54–2.99	1.04	0.34–2.08	1.31	0.00–2.62
Serowe	1.00	-	1.00	-	1.00	-
**Religion**						
None	1.00	-	1.00	-	1.00	-
Other	0.96	0.32–1.82	0.54	0.32–1.22	1.80	0.12–4.76
Pentecostal	1.17	0.54–3.01	1.33	0.23–2.66	2.03	0.51–4.96
Protestant	2.31	0.27–4.62	3.41	0.79–6.82	13.2	0.81–26.4
Traditional	1.05	0.35–2.10	2.36	0.32–4.72	6.56	0.61–13.2
**Education level**						
Completed primary	0.07	0.01–1.11	2.09	0.56–4.18	5.69	0.51–11.5
Completed secondary	1.64	0.62–3.02	1.23	0.17–3.46	0.09	0.02–3.11
Completed tertiary	0.96	0.32–1.82	5.42*	2.03–10.8	0.02	0.00–1.91
None	1.01	0.31–2.03	4.67	0.71–9.57	0.71	0.11–1.44
Not completed primary	1.05	0.35–2.10	1.17	0.53–3.00	0.27	0.07–3.17
Not completed secondary	1.08	0.38–2.12	1.29	0.61–5.13	0.03	0.00–3.23
Not completed tertiary	0.94	0.30–1.80	2.82	0.68–6.14	1.23	0.51–3.05
**Occupation**						
Clerical and/or management and/or administrative	1.00	-	1.00	1.00	1.00	1.00
Crafts	7.26	0.95–14.5	4.76	0.74–9.58	3.04	0.78–6.08
Farmer	6.65	0.93–13.3	2.29	0.13–7.15	0.38	0.02–3.64
Housewife	7.37	0.97–14.5	5.22	0.43–10.4	1.37	0.23–3.73
None	7.13	0.93–4.6	3.72	0.24–7.36	15.7	0.91–31.4
Other	3.52	0.32–7.12	10.3	0.98–20.6	1.11	0.47–4.22
Professional technical	2.41	0.61–5.17	4.95	0.53–5.80	0.05	0.00–1.03
Trader	1.38	0.25–3.75	1.35	0.23–7.50	0.07	0.00–3.13

BC, breast cancer; AOR, adjusted odds ratio; CI, confidence interval.

* , *p*-value of differences between male and female;

** , implies the differences observed in knowledge of risk factors between male and female are statistically significant (highly reliable).

The odds of knowledge about breast cancer were significantly higher among individuals who resided in the Francistown district than in Serowe. For instance, people residing in Francistown were more likely to be knowledgeable about breast cancer (AOR = 2.73, CI = 1.12–3.78), its symptoms (AOR = 3.08, CI = 1.52–7.16) and risk factors (AOR = 2.40, CI = 1.02–4.80) compared to those residing in Serowe. Meanwhile, knowledge about risk factors was also significantly higher among individuals residing in Goodhope (AOR = 2.37, CI = 1.01–4.74) than in Serowe. For education, the odds of knowledge about breast cancer symptoms were significantly higher among individuals who had completed tertiary education (AOR = 5.42, CI = 2.03–10.8) compared to those with lower-level educational qualifications.

## Discussion

Our study uncovered compelling insights crucial for shaping breast cancer prevention programmes in Botswana. The gender imbalance among outpatients in our study, with 30.7% male and 69.3% female, aligns with other studies in Botswana (Khuwa, Matlala & Ntuli 2022; Statistics Botswana 2018). This suggests a female-dominated study group, reflecting gender disparities in health-seeking behaviour likely influenced by illness patterns, socio-cultural norms, and healthcare access. Efforts must address barriers preventing males from seeking healthcare, such as enhancing service accessibility, tackling social stigma, and promoting health literacy.

Most participants viewed breast cancer as a serious illness in Botswana and believed it to be hereditary, recognising that early detection could improve survival rates. Females demonstrated significantly greater knowledge about breast cancer, its symptoms, and risk factors compared to males. Nearly all participants were aware of breast cancer, though only half acknowledged that males can also get cancer, echoing another study where 61.1% of participants were unaware of male breast cancer (Altiner et al. 2023). Male breast cancer is significantly rarer, making up less than 1% of all cases, due to biological differences (Akbari, Nasiri & Ziaee 2024). While rare, increased awareness among males can lead to earlier detection and better outcomes. Education for both genders is crucial for informed health decisions, mutual support, and correcting misconceptions about gender-specific risks.

Understanding breast cancer is essential for both male and female, aiding in prevention and early detection. A lack of general knowledge about breast cancer and its risk factors can increase morbidity and mortality (WHO 2024). Almost all respondents, regardless of gender, had a sound general understanding of breast cancer. Similar trends were observed in Lesotho (Ramathebane et al. 2022). However, prior to diagnosis, survivors in Botswana (Mbuka-Ongona & Tumbo 2013), Saudi Arabia (Farsi, Al-Wassia & Merdad 2020) and South Africa (Ramathuba, Ratshirumbi & Mashamba 2015) exhibited poor knowledge. Other studies reported low knowledge levels among newly diagnosed females in Namibia (15%), Zambia (15%), Uganda (25%) and Nigeria (25%) (McKenzie et al. 2018). This persistently low level of knowledge of breast cancer risk factors may be compounded by a lack of robust screening infrastructure, which limits opportunities for early detection and, consequently, reduces community exposure to educational information about risk factors.

Despite general awareness, both genders showed poor understanding of breast cancer risk factors and symptoms. Less than 5% correctly identified poverty, advanced age, late childbearing, early menarche, late menopause, and oral contraceptive use as risk factors. Between 5% and 15% recognised high-fat diets, alcohol, tobacco use, obesity, and family history as risk factors. This knowledge gap highlights missed opportunities for preventive care and early diagnosis. Such disparities may be deeply rooted in cultural barriers that discourage open discussion about breast cancer, alongside limited educational access to comprehensive, tailored sexual and reproductive health information. Bridging this gap is vital for promoting early detection and improving outcomes as recommended by the WHO (2024).

While poverty exacerbates late-stage diagnosis and lowers survival rates due to inadequate healthcare access, there is a misconception linking wealth with better healthcare access (Chen et al. 2024; Moss et al. 2020, 2022). In some African countries, wealthier individuals favour processed over traditional foods, raising their breast cancer risk (Reardon et al. 2021). This dietary shift, despite healthcare access, increases breast cancer risk, challenging the notion of wealth as a health advantage (Nouri et al. 2024). A synthesis of these findings indicates that monolithic, ‘one-size-fits-all’ paradigms in oncological prevention and therapeutic management yield suboptimal clinical outcomes. These findings collectively advocate for a paradigm shift towards stratified, precision-based interventions, arguing that addressing specific socioeconomic determinants of health, population heterogeneity, and contextual environmental factors is necessary to enhance efficacy.

Research emphasises the importance of identifying predictors of health conditions for designing interventions (Kent et al. 2020). Our study identified gender, age, education, marital status, and location as predictors of breast cancer knowledge. Females had significantly better knowledge, consistent with other studies (Adewale Adeoye 2023). Moreover, many males were poorly informed and held negative attitudes towards females with breast cancer, potentially impeding support and overall care as reported in other studies (Ozaydin & Bozdogan 2020). Educating males in patriarchal societies like Botswana can significantly impact women’s healthcare decisions.

Our study revealed that participants aged 18 years – 24 years had greater breast cancer awareness levels, likely due to better information access and educational campaigns. Botswana’s strategic placement of non-communicable disease interventions in the Office of the President reflects a commitment to prioritising resources and increasing access to information for younger demographics. Additionally, civil society organisations are actively increasing access to breast cancer information to younger age groups in tertiary institutions. Hence, early detection and tailored strategies for younger females are essential, given the reported cases among this population group (Ntekim, Oluwasanu & Odukoya 2022).

Higher education emerged as a strong predictor of breast cancer knowledge, with tertiary-educated participants demonstrating better awareness of symptoms (Manouchehri et al. 2022; Mertens et al. 2023). Botswana’s high literacy rate, 88.01% for females and 88.93% for males (Statistics Botswana 2015), supports this finding, aligning with research showing educated people have a better grasp of risk factors and symptoms (Awogbayila et al. 2023; Effiong 2023; Effiong, Afolabi & Chinedu 2024; Osei-Afriyie et al. 2021). As educated people are more knowledgeable about breast cancer risk factors, the country should integrate breast cancer education into school curricula and ensure that the entire citizenry has access to education. This presents an opportunity for the country to raise breast cancer awareness and promote regular screening and early diagnosis.

Participants in relationships had better knowledge of breast cancer symptoms than those widowed, highlighting the role of social support, consistent with other studies (Yang et al. 2022). This finding underscores the need to involve partners in awareness campaigns, enhancing social support and quality of life for patients. Community-based educational interventions play a pivotal role in raising awareness. Our study found that individuals in Goodhope had better knowledge of breast cancer risk factors, likely due to prior initiatives by organisations like Jhpiego. Similarly, communities in Francistown benefited from ongoing educational activities by Non-Governmental Organisations (NGOs) such as Journey of Hope Botswana. These interventions significantly improved knowledge, fostering better understanding and outcomes. This aligns with global evidence showing community interventions enhance breast cancer awareness (Akinboye et al. 2023; Barrow et al. 2024; Sarker et al. 2022). Therefore, low knowledge level of breast cancer risk factors is not merely a lack of interest, but often a result of systemic, educational, and cultural barriers that must be addressed through targeted, community-based interventions.

### Strengths

One strength of our study was the use of structured questionnaires adapted from the Breast Cancer Initiative (Dvaladze et al. 2016), facilitating comparable results across studies and leveraging validated tools. Conducted across various districts, the study provided insights into health behaviours. Including males as participants deepened our understanding of gender dynamics, societal norms, and breast cancer’s impact, informing interventions to improve women’s access to care and challenge harmful perceptions.

### Limitations

While patient exit interviews offer valuable perspectives, they do not capture the experiences of those not seeking healthcare in these settings. The findings should be interpreted within the context of these limitations.

### Implications and recommendations

The finding that most participants are familiar with ‘breast cancer’ but lack knowledge about its risk factors and symptoms has significant public health implications. This gap calls for targeted educational interventions to enhance understanding, potentially leading to earlier detection and improved outcomes. Future research should focus on developing and evaluating interventions that address these knowledge gaps in Botswana.

## Conclusion

This study assessed the knowledge of breast cancer, its symptoms, and risk factors among adult males and females attending outpatient departments at four oncology-designated public health facilities across Botswana. This study reveals a sharp contrast regarding knowledge of breast cancer and its risk factors in Botswana. While most participants had heard of breast cancer, their understanding of risk factors and symptoms was limited. Our data show that factors like age, gender, marital status, education level and location largely dictate who gets left behind, with males and older residents facing the steepest knowledge gaps. The study highlights the need for targeted strategies that address cultural stigmas and actively bring males into the conversation. Through the integration of breast cancer education into school lessons and scaling up local outreach programmes, like the successful pilots in Goodhope and Francistown, the country can build a more breast cancer-literate society. Equipping individuals with the knowledge and tools for informed decisions about the entire breast cancer cascade can improve early detection and survival rates, aligning with Sustainable Development Goal 3, aimed at reducing premature mortality from non-communicable diseases by one-third by 2030.

### Contribution

Our study adds to the literature on the importance of moving beyond general campaigns towards targeted education that gives people the knowledge and tools they need to take charge of their health and seek medical help when they need it. By identifying health systems and cultural barriers as key reasons for low awareness, this study offers practical evidence for policymakers. This can help them prioritise specific actions, making our findings more useful for managing breast cancer in Botswana.
